# List of Diseases from the 1st of January, to the 10th of February, 1799: Being the Result of the Public and Private Practice of a Physician at the West End of London

**Published:** 1799-03

**Authors:** 


*Liſt of Diſeaſes from the 1ſt of January, to the 10th of February, 1799: being the Reſult of the public and private Practice of a Phyſician at the Weſt End of London.*

| | |
|---|---|
| ACUTE OR FEBRILE DISEASES. | |
| Catarrh . . . . | 33 |
| Peritoneal Inflammation . | 1 |
| Acute Rheumatiſm . . | 29 |
| Ophthalmia . . . . | 4 |
| Inflammatory Sore-throat . | 4 |
| Ulcerated, or ſpecky Sore-throat | 6 |
| Scarlatina Anginoſa . . . | 5 |
| Meaſles . . . . | 3 |
| Small-pox . . . . | 4 |
| Hooping-cough . . . | 7 |
| Malignant Fever . . . | 5 |
| Slow Fever . . . . | 4 |
| Hectica . . . . | 3 |
| Child-bed and Milk Fever . | 4 |
| Acute Diſeaſes of Infants . . | 12 |
| Quotidian . . . . | 2 |
| CHRONIC DISEASES. | |
| Cough and Dyſpnœa . . | 86 |
| Pulmonary Conſumption . . | 17 |
| Spitting of Blood . . . | 5 |
| Epiſtaxis . . . . | 2 |
| Chronic Rheumatiſm . . | 6 |
| Rheumatic Tooth-ach . . | 4 |
| Sciatica . . . . | 3 |
| Dropſy . . . . | 7 |
| Aſthenia . . . . | 19 |
| Cephalæa and Vertigo . . | 17 |
| Syncope . . . . | 1 |
| Palſy . . . . . | 6 |
| Chorea Sancti Viti . . | 2 |
| Epilepſy . . . . | 1 |
| Hyſteria . . . . | 2 |
| Palpitatio . . . . | 3 |
| Hydrocephalus . . . | 1 |
| Dyſpepſia . . . . | 15 |
| Gaſtrodynia . . . | 8 |
| Hæmorrhoids . . . | 2 |
| Enterodynia . . . | 10 |
| Diarrhœa . . . . | 10 |
| Colica Pictonum . . . | 2 |
| Hernia . . . . | 2 |
| Gravel . . . . | 3 |
| Enureſis . . . . | 2 |
| Hæmaturia . . . . | 1 |
| Iſchuria . . . . | 4 |
| Menorrhagia . . . | 5 |
| Chloroſis and Amenorrhea . | 5 |
| Fluor Albus . . . | 2 |
| Scirrhus Uteri . . . | 1 |
| Scirrhus Hepatis . . . . | 2 |
| Scrophula . . . . | 8 |
| Contractura . . . . | 1 |
| Tabes Meſenterica . . . | 2 |
| Worms . . . . | 6 |
| Dentition . . . . | 3 |
| Thruſh . . . . . | 4 |
| Papulous Eruptions on the Skin | 3 |
| Lepra . . . . . | 1 |
| Scaly Tetter . . . | 3 |
| Prurigo . . . . . | 1 |
| Itch . . . . . | 4 |
| Scalded Head . . . | 6 |
| Ecthyma . . . . . | 3 |
| Impetigo . . . . | 1 |
| Shingles . . . . . | 1 |
| Pemphigus Infantilis . . . | 1 |
| Erythema . . . . | 2 |
| Gutta Roſea . . . . | 2 |

